# Extended Neural Metastability in an Embodied Model of Sensorimotor Coupling

**DOI:** 10.3389/fnsys.2016.00076

**Published:** 2016-09-23

**Authors:** Miguel Aguilera, Manuel G. Bedia, Xabier E. Barandiaran

**Affiliations:** ^1^Department of Computer Science and Systems Engineering, University of ZaragozaZaragoza, Spain; ^2^Department of Psychology, University of the Balearic IslandsPalma de Mallorca, Spain; ^3^ISAAC Lab, Aragon Institute of Engineering Research, University of ZaragozaZaragoza, Spain; ^4^Department of Philosophy, University School of Social Work, University of the Basque Country (UPV/EHU)Vitoria-Gasteiz, Spain; ^5^Department of Logic and Philosophy of Science, IAS-Research Center for Life, Mind and Society, University of the Basque Country (UPV/EHU)Donostia - San Sebastián, Spain

**Keywords:** neural assemblies, metastability, criticality, synaptic plasticity, embodied cognition, sensorimotor coupling, evolutionary robotics

## Abstract

The hypothesis that brain organization is based on mechanisms of metastable synchronization in neural assemblies has been popularized during the last decades of neuroscientific research. Nevertheless, the role of body and environment for understanding the functioning of metastable assemblies is frequently dismissed. The main goal of this paper is to investigate the contribution of sensorimotor coupling to neural and behavioral metastability using a minimal computational model of plastic neural ensembles embedded in a robotic agent in a behavioral preference task. Our hypothesis is that, under some conditions, the metastability of the system is not restricted to the brain but extends to the system composed by the interaction of brain, body and environment. We test this idea, comparing an agent in continuous interaction with its environment in a task demanding behavioral flexibility with an equivalent model from the point of view of “internalist neuroscience.” A statistical characterization of our model and tools from information theory allow us to show how (1) the bidirectional coupling between agent and environment brings the system closer to a regime of criticality and triggers the emergence of additional metastable states which are not found in the brain in isolation but extended to the whole system of sensorimotor interaction, (2) the synaptic plasticity of the agent is fundamental to sustain open structures in the neural controller of the agent flexibly engaging and disengaging different behavioral patterns that sustain sensorimotor metastable states, and (3) these extended metastable states emerge when the agent generates an asymmetrical circular loop of causal interaction with its environment, in which the agent responds to variability of the environment at fast timescales while acting over the environment at slow timescales, suggesting the constitution of the agent as an autonomous entity actively modulating its sensorimotor coupling with the world. We conclude with a reflection about how our results contribute in a more general way to current progress in neuroscientific research.

## 1. Introduction

Generally, neurodynamic approaches have focused in understanding what kind of neural organization is necessary to cope with the requirements of an external world. Assuming that the brain is subject to demanding conditions from its environment, the challenge is to explain what type of neural computation or what form of organization of neural spatiotemporal patterns might be capable of satisfying the requirements for adaptive, conscious, cognitive activity. This has led to progress in the definition of a framework able to account for the brain's ability to display a rich set of meaningful behaviors. Nowadays, a popular view in neuroscience holds that the human brain is structured into a large number of areas in which information is highly segregated into local clusters and, at the same time, functionally integrated (Damasio, 1989; Varela, 1995; Tononi and Edelman, 1998). One of the most plausible mechanisms hypothesized to be behind this equilibrium between integration and segregation is metastable phase locking between neural assemblies over multiple frequency bands. This mechanism has been proposed to explain how the brain flexibly enters and exits coherent spatiotemporal patterns of neural activity (Kelso, 1995; Varela et al., 2001; Le Van Quyen, 2011). Subsequently, the notion of metastable neural assemblies as building blocks of brain organization has become relatively widespread in large-scale neuroscience studies (e.g., Werner, 2007a; Buzsáki, 2010; Edelman et al., 2011; Ward, 2011).

Nevertheless, when analysing and modeling brain organization, a crucial aspect of cognitive dynamics is frequently neglected: the sensorimotor coordination that continuously feeds back into brain dynamics (from saccadic eye movements to proprioception; from perception to action; O'Regan and Noë, 2001; Aguilera et al., 2013; Engel et al., 2013). Mental processes such as perception, emotion or intention are not limited to neural processes inside the brain, but produced through a flexible integration of the dynamics of brain, body and environment in a distributed manner. Hypotheses addressing this issue propose that brain organization consists in a plastic system of open loops developed in the process of life and closed to full functional cycles in every interaction with the environment (Fuchs, 2011), being the role of the central nervous system to transform and diversify these loops. In addition, it has been proposed that the behavior neural tissue in isolation might be restricted to little more than exhibiting spontaneous synchronization and other behaviors common to nonlinear dynamical systems, and the brain may operate as a *metastable circuit breaker* flexibly switching between different dynamic fields of agent-environment engagement (Dotov, 2014).

**Figure 1:**
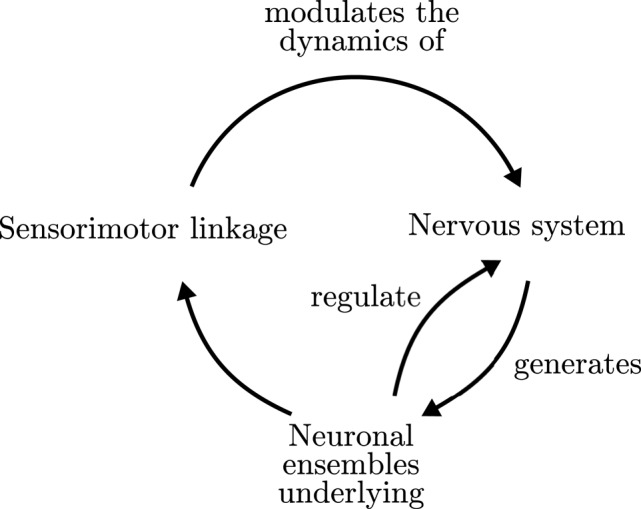
**Organizational closure of the nervous system**. Adapted from Varela (1997).

Furthermore, enactive approaches to neurodynamics have proposed that the formation and dissolution of neural assemblies in the brain must be embedded in sensorimotor regulatory cycles, producing the emergence of global organism-environment processes, which in turn affect their constituent elements (Thompson and Varela, 2001; Varela and Thompson, 2003; Di Paolo et al., 2016). One of the central contributions to this issue has been the notion of an operational closure of the nervous system (Varela, 1997; Di Paolo and Thompson, 2014), illustrated in Figure 1. Operational closure implies a circular regulation in which the coordinated activity of the neural system gives rise to the emergence of neural ensembles (or “cell assemblies”; Hebb, 1952), driving the behavior of the organism, which in turn generates a sensory input into the neural system closing a double regulatory loop. According to Francisco Varela,

The nervous system is organized by the operational closure of a network of reciprocally related modular subnetworks giving rise to ensembles of coherent activity such that: (i) they continuously mediate invariant patterns of sensorymotor correlation of the sensory and effector surfaces; (ii) give rise to a behavior for the total organism as a mobile unit in space. The operational closure of the nervous system then brings forth a specific mode of coherence, which is embedded in the organism. This coherence is a cognitive self: a unit of perception/motion in space, sensorymotor invariances mediated through the interneuron network (Varela, 1992, p.10).

Nevertheless, there are a lack of good models or precise characterizations of the operational closure of the nervous systems and the kind of interaction that takes place between neural, bodily and sensorimotor cycles (Barandiaran, 2016). Thus, it is far from clear how to characterize this sensorimotor specific form of coherence and how is it constitutive of cognitive activity. Clarifying this issue is of fundamental importance for embodied neurodynamic views in order to propose solid explanatory alternatives to internalist perspectives of brain organization.

In the case of metastability in cognitive processes, we know that metastable behavior is not restricted to the brain, but also extends to behavioral patterns (Kelso, 1995; Kelso et al., 1995). Nevertheless it is not clear what the relation is between behavioral metastability and underlying neural metastability. Consider, for example, the case of perceiving an ambiguous image (e.g., an image perceived either as a face or as meaningless shapes, or a Necker cube which can be alternatively seen as oriented in two different positions) in which perception can alternatively and spontaneously switch from one mode to another. If we take a look at the brain we can observe the emergence of transient assemblies of neural synchronization when one mode of perception arises (Rodriguez et al., 1999), and at the same time if we were to analyse the switching of perceptive patterns the subject is engaged in we can observe signatures of metastable behavior (Kelso et al., 1995, Section 3). This raises questions around whether long term behavioral metastability is the product of a direct mapping of intrinsic metastable states of the brain into behavior or, conversely, metastability of both the brain and behavior is a product of the whole brain-body-environment system coupled by sensorimotor processes (the interaction of neural dynamics, retinal activation, patterns of saccadic eye movements, etc.). These are difficult questions, requiring comparison between processes taking place at different scales (neurodynamic, sensorimotor, conductual, etc.) that pose important experimental difficulties. In the past, evolutionary robotics has been a highly productive tool for finding non-intuitive solutions to complex problems and understanding many-to-many relations between different scales of behavior (Nolfi and Floreano, 2000; Harvey et al., 2005). In the same tradition, in this paper we present an artificially evolved agent to explore the relation between neural and behavioral metastability in a simple bistable task. We choose a phototactic task^1^ in which the agent alternatively develops a preference between two types of light (e.g., two different colors) as an example of a simple task involving metastable neural and behavioral dynamics.

Our hypothesis is that the slow modulation of synaptic plasticity over the sensorimotor coupling increases and sustains the metastability of neuro-behavioral integrated states in a manner that cannot be reduced to the dynamics of the brain in isolation, nor the brain receiving a structured input. These integrated metastable states are associated with specific modes of coherence in neural structures when they are engaged in bodily and environmental processes, as the brain-body-environment system becomes an operationally closed entity. The specificity of these modes of coherence is hypothesized to be related to the particularities of autonomous agency and the operational closure of the nervous system, i.e., the characteristics that allow us to describe an agent as an individual entity albeit in continuous interaction with its environment: such as the self-constitution of the agent as an entity or an asymmetrical interaction with its world in which it actively modulates its own sensorimotor coupling (see Barandiaran et al., 2009). Here, we propose a minimalistic approach to address some of the difficult questions arising from these ideas. We introduce a robotic model equipped with just three oscillatory units and synaptic plasticity in their connections. Because of its simplicity, this approach allows us to tackle the problem with a system about which we have complete knowledge and that is tractable using dynamical systems techniques of analysis.

In the following sections we first introduce the robotic agent and an artificial evolution process to obtain a model displaying metastable behavioral patterns in a bistable phototactic task. Then, we describe our methodology for analysing the role of the sensorimotor loop in the generation of metastable behavioral patterns, combining (1) the comparison of a *situated* agent interacting directly with its environment and a *passively-coupled* agent which is fed a signal identical to the one received by the situated agent, but it cannot influence its environment, and (2) a statistical description of the states of the agent and the environment together with the use of different tools from information theory to quantify the metastability in its behavior and the interaction between different scales of description of the robot (oscillatory activity, synaptic plasticity and behavioral patterns). In this framework we perform experiments showing that (1) the bidirectional coupling between agent and environment brings the system closer to a regime of criticality and triggers the emergence of additional metastable states, which are not found in the brain in isolation but extended to the whole system of sensorimotor interaction, (2) the synaptic plasticity of the agent is key to sustain open structures in the neural controller of the agent flexibly engaging and disengaging different behavioral patterns that sustain sensorimotor metastable states, and (3) this creates an asymmetrical circular loop of interaction between agent and environment, in which the agent is able to respond to variability of the environment at small timescales, while acting over the environment at large timescales. We conclude that metastability of neural dynamics can be extended to sensorimotor metastable states and that, in our model, this takes place when the agent establishes a specific circular relation with its environment, suggesting that the extension of metastable dynamics from the brain to interactive behavioral patterns is connected with specific forms of engagement with the world characteristic in autonomous agency.

## 2. Methods

As we proposed above, our goal is to explore the relation between metastability in brain dynamics and behavior in a robotic model in order to test the hypothesis that some behavioral metastable states cannot be reduced to brain dynamics alone and are instead the product of an integration of brain, bodily and environmental dynamics. In order to do this, we design a model with the ability of presenting flexibility in both neural and behavioral patterns (which will be evolved using a genetic algorithm in order to reduce the constraints imposed onto the model) and we propose a framework of analysis allowing us to characterize metastable states and relations between components of the model, as well as a comparison of the behavior of the brain with and without the effect of the sensorimotor loop in equivalent conditions.

### 2.1. Model of a neurodynamic controller with relational homeostasis embedded in a robotic agent

We propose a model of homeostatic adaptation inspired by previous work in evolutionary robotics (Iizuka and Di Paolo, 2007; Di Paolo and Iizuka, 2008), defining an adaptive mobile agent controlled by a plastic oscillatory neural system. This model is not intended to represent the activity of individual neurons but, more generally, to capture the dynamics of neural oscillations at a mesoscopic level, where integration mechanisms are hypothesized to be based on phase synchronization processes between neuronal groups (Varela et al., 2001), thus representing large-scale synchronization of brain regions that are anatomically far apart. Since the model is described in detail in Aguilera et al. (2015), we provide here a brief description.

**Figure 2:**
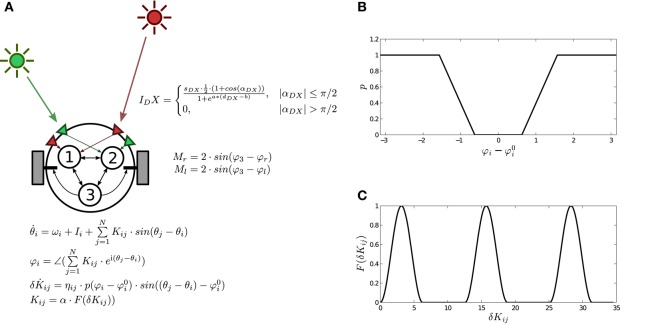
**The robotic agent with three plastic oscillatory units. (A)** Schema of the agent, the environment, sensors and motors, and the neural controller. **(B)** Plastic function $p\left(\Phi_{i}-\Phi_{i}^{0}\right)$, in which plasticity depends on the difference between the weighted phase relation Φ_*i*_ of the neural oscillator *i* with respect to other oscillators and the preferred weighted phase relation $\Phi_{i}^{0}$. **(C)** Mapping function *F*(δ*K*_*ij*_) which transforms weight values δ*K*_*ij*_ into the actual value of coupling strengths between oscillators *K*_*ij*_. Reproduced from Aguilera et al. (2015).

The agent incorporates a neural controller defined as a fully connected Kuramoto network (Acebrón et al., 2005) with three units defined as:

(1) $$\begin{array}{l} {\dot{\theta }}_{i}=\omega_{i}+I_{i}+\overset{N}{\underset{j=1}{\sum }}K_{ij}\cdot sin\left(\theta_{j}-\theta_{i}\right) \end{array}$$

where θ_*i*_ represents the phase of oscillator *i*, ω_*i*_ is its natural frequency (range [0, 5]), *K*_*ij*_ is the coupling strength between oscillators *i* and *j*, and *I*_*i*_ represents the sensory inputs. The behavior of the neural controller is modulated by plastic mechanisms preserving phase relational invariances of the system, defined as:

(2) $$\begin{array}{l} \delta {\dot{K}}_{ij}=\eta_{ij}\cdot p\left(\Phi_{i}-\Phi_{i}^{0}\right)\cdot sin\left(\left(\theta_{j}-\theta_{i}\right)-\Phi_{i}^{0}\right) \end{array}$$

where δ*K*_*ij*_ are the connection weights, η_*ij*_ is the rate of plastic change (range [0, 0.9]) of each connection, and Φ represents the phase difference of oscillator *i* with respect to the sum of the oscillators connected to it weighted by the strength of their connections:

(3) $$\begin{array}{l} \Phi_{i}=∠\left(\overset{N}{\underset{j=1}{\sum }}K_{ij}\cdot e^{i\left(\theta_{j}-\theta_{i}\right)}\right) \end{array}$$

where ∠ denotes the phase of a complex value and *i* is the imaginary unit. $\Phi_{i}^{0}$ (range $\left[\frac{-\pi }{2},\frac{\pi }{2}\right]$) stands for the preferred phase relation of the oscillatory node. Finally, the function *p*(*x*) determines the level of plastic change for all incoming weights of a node, which is activated when the value of Φ_*i*_ is far from $\Phi_{i}^{0}$ (Figure 2B). When plastic changes take place, connection strengths change following a continuous non-monotonic function *K*_*ij*_ = α · *F*(δ*K*_*ij*_) (Figure 2C) designed to explore the full configuration space^2^, where α is a constant (range [0, 5]) that regulates the coupling strength.

In short, the model works under the assumption that large-scale neural oscillatory components try to maintain a preferred phase relation with respect to other oscillatory components by means of plastically regulating the strength of their connectivity. The model is designed to present the possibility of metastable behavior at different states. Kuramoto oscillator networks can display metastable states when the connection strengths are below a critical point of complete synchronization. Also, we defined the evolution of synaptic plasticity in such a way that the agent can potentially explore all different available possibilities of engagement with the environment, and furthermore be able to exploit forms of behavioral metastability.

The agent is modeled as a simulated wheeled robot with a circular body of radius 4 and two diametrically opposed motors (Figure 2A), driving the agent backwards and forwards. The agent's mass is assumed to be small enough for inertial resistance to be negligible, thus its translational speed is calculated as the vectorial average of the motor velocities, and the angular speed as the difference of the motor velocities divided by the body diameter. Motor outputs are calculated from the phase relation Φ_3_ of the effector oscillator, with a gain parameter of value 2:

(4) $$\begin{array}{l} \begin{array}{c} M_{r}=2\cdot sin\left(\Phi_{3}-\Phi_{r}\right)\\ M_{l}=2\cdot sin\left(\Phi_{3}-\Phi_{l}\right) \end{array} \end{array}$$

where Φ_*r*_ and Φ_*l*_ (range [0, 2π]) are bias terms which map the motor output into the actual motor activation.

The agent has two pairs of sensors (right and left) for each of the different light sources *A* and *B*. Each sensor points to a direction at π/3 radians from the forward direction. Light *A* sensors are connected to oscillator 1 and light *B* sensors are connected to oscillator 2. The effects of both the angle and the distance on the sensor activation are represented by the following function:

(5) $$I_{DX}=\{\begin{array}{ll} \frac{s_{DX}\cdot 0.5\cdot (1+cos(\alpha_{DX}))}{1+e^{a\cdot (d_{DX}-b)}}, & |\alpha_{DX}|\leq \pi /2\\ 0, & |\alpha_{DX}|>\pi /2 \end{array}$$

where *X* can represent either light *A* or *B*, *D* stands for either right or left sensor, α_*DX*_ is the angle of sensor *DX* to light *X*, *d*_*DX*_ is the distance between sensor *DX* and light *X*, and *a* and *b* have the arbitrary values of 0.03 and 100 respectively. The light intensity received at each sensor is multiplied by a gain parameter *s*_*DX*_ (range [−8, 8]), feeding the resulting value to the corresponding oscillator's input *I*_*i*_. A full schema of the robot is represented in Figure 2A.

All parameter values (except where otherwise specified) are fixed by a genetic algorithm within the indicated range. A population of 20 agents is evolved using a rank-based genetic algorithm with elitism. Each of the agent parameters ω_*i*_, *s*_*DX*_, α, η_*ij*_, Φ_*r*_, Φ_*l*_ and $\Phi_{i}^{0}$ is encoded into a 5 bits string representing real numbers uniformly distributed within the specified range. For each generation, the best 4 agents (20% of the population) pass to the next generation without change. For the remaining slots, pairs of individuals are selected for crossover with a probability proportional to their fitness value, and new individuals are created mixing their genes (bit series) by adding a mutation probability of 3% for each gene.

The agents are evolved for displaying a metastable behavior in which the agent has to develop switching preferences toward two different types of light. That is, there is an environment with two types of light (e.g., two colors) and we want the agent to develop a preference toward one of them (e.g., repeatedly interacting with it) while being able to switch its preference to the other light depending on its internal configuration. This behavior is chosen because it demands the agent to present a robust phototactic behavior while at the same time presenting flexibility in the creation and dissolution of behavioral preferences. An evaluation procedure is proposed for the genetic algorithm in order to accomplish this objective, consisting in four different tasks designed by Iizuka and Di Paolo (2007): a single light *A*, a single light *B*, one light *A* and a blinking light *B*, one light *B* and a blinking light *A* (blinking lights illuminate with a probability of 0.15 for each time step). The agent gains fitness by approaching the non-blinking light. The objective of this configuration is to create a “dummy” that encourages the agent to learn to ignore one of the lights while approaching the other. Lights appear at a random distance, [100, 150]. When two lights are present, they appear, from the agent's point of view, with a random separation within the range [π/2, 3π/2]. The length of each trial is 125*s*.

Each individual agent is tested for 12 independent runs (3 for each of the 4 tasks) consisting of a series of trials where a light or a pair of lights are presented to the agents for a fixed time. Synaptic weights δ*K*_*ij*_ are reset to initial random values before each run. Each run consist of 8 trials in which the agent is presented with one or two lights for a specified time. Only the last 3 trials of each run are evaluated in order not to penalize slow plastic changes and bootstrap evolution. All simulations are run with an Euler step of 0.1.

Fitness for each trial is calculated in three terms, *F*_*trial*_ = (*F*_*D*_ + *F*_*p*_) · *F*_*H*_. *F*_*D*_ = 1 − *d*_*f*_/*d*_*i*_, where *d*_*f*_ and *d*_*i*_ respectively correspond to the final and initial distances to the target light. *F*_*p*_ is equal to the proportion of time that the agent spends within a distance of less than 4 times its body radius (i.e., a distance of 16) to the target light during a trial. *F*_*H*_ represents the mean level of homeostasis in the system, computing the mean degree of homeostasis $\frac{1}{3}\underset{i}{\sum }\left(1-p\left(\Phi_{i}-\Phi_{i}^{0}\right)\right)$ (i.e., 1 minus the level of plasticity) for each oscillator. The genetic algorithm is run for 500 generations, reaching a stable level of fitness around 0.45. The best performing agent from the last generation (which is able to reach both lights ignoring the dummy) is selected. The code simulating the behavior of the agent and the parameters obtained from the genetic algorithm can be accessed from the following repository https://github.com/IsaacLab/HNA-robotic-model/tree/master/minimal-preference-task.

### 2.2. Conceptual setup for testing sensorimotor integration: situated vs. passively-coupled agents in an open environment

In order to explore how the agent exploits internal and sensorimotor metastability we simulate the agent in an open environment (which was never experienced during training) in which the agent can develop sustained preferences toward the two types of light. In this case, the two lights are always present with equal intensities, and a new pair of lights is generated periodically after a given time (starting a new trial). The best performing agent from the last generation of the genetic algorithm agent is simulated in a virtual environment which presents a series of pairs of lights, giving the agent a time of 1250 steps to choose and approach one of them. As analysed elsewhere (Aguilera et al., 2015), the agent is able to develop stable preferences toward one of the lights, maintaining it for several trials until the preference is changed. The switching of preferences depends on the long-term interaction between internal plastic mechanisms and the encountered configurations of the environment. Different neural cell assemblies arise connected with particular patterns of behavior of the agent, and at slower timescales synaptic plasticity modulates the emergence and dissolution of these behavioral patterns. A video of the behavior of the agent (including plastic mechanisms) can be found at https://vimeo.com/53847420.

Once defined the agent in which we want to explore the emergence of metastable behavioral patterns, we propose a sensorimotor null model to be compared with our model in order to test the influence of the sensorimotor loop in the generation of metastable states in the agent. To this end, we propose an agent which maintains the structure of the received input but presents a disrupted coordination with its environment. Thus, we will compare:

1. A *situated* agent with normal sensorimotor interaction.
2. A *passively-coupled* agent, in which the input fed to the agent is recorded from the behavior of the situated agent. Thus, in the passively-coupled agent the received input is decoupled from the activation of the motors but it maintains an structure as if it was generated by real interaction.

With this comparison we can detect the effects that, despite being the result of sensorimotor coordination, cannot be replicated just by using an input with an adequate structure. This is a subtle difference, but if genuine sensorimotor coordination is constitutive of a cognitive process, the same process should not take place when the agent is passively processing an input with the rig as an input-structured process.

### 2.3. Discretization and probability density function of the system

In the experimental setup defined above, we will use information theory tools in order to get a better understanding of how the different elements of the neural controller and the agent's behavior interact, using a symbolic representation of the system states. Understanding the coordination between neural ensembles and sensorimotor activity is not trivial due to the moderately high dimensionality of the system (9 dimensions of the neural controller, plus the dimensions of body and environment). Nevertheless, the system can be simplified by reducing both the state of neural ensembles and synapse configurations to discrete values representing the state of a network, plus a binary variable representing the behavior of the agent (reaching one light or the other).

**Figure 3:**
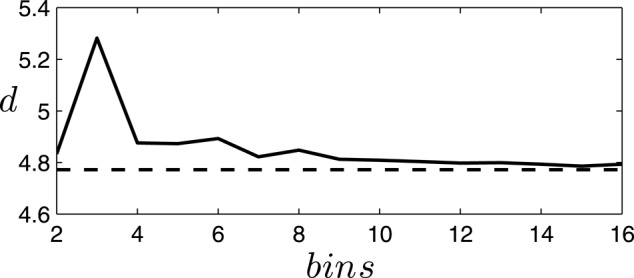
**Increment of dimensionality of the discretized system for different bin choices**. The dimensionality of the data generated by the situated agent (the passively-coupled agent yields similar results) with continuous (dashed line) or discrete (continuous line) values using different numbers of equally spaced bins in the discretization. We observe how a binary description (2 bins) is a good discretization of the system, presenting a small increase in dimensionality only matched by discretizations with 7 or more bins. A discretization with 3 bins is particularly unfit since it cannot capture the covariance of small fluctuations around the central bin.

To simplify the analysis, we are interested in a description of the system minimizing the number of symbols needed to describe the states of the robot, while maintaining the properties of the system. In this case, we find that a binary discretization is a good choice for describing the system. In order to ascertain a good discretization of the system, we test the validity of different possible discretizations by comparing the dimensionality of the original and discretized data through principal component analysis on their covariance matrix. We use the following definition of dimensionality (Abbott et al., 2009):

(6) $$d={\left(\sum_{i=1}^{N}{\tilde{\lambda }}_{i}{}^{2}\right)}^{-1}$$

where $\tilde{\lambda_{i}}$ are the normalized eigenvalues of the covariance matrix expressing the fraction of the variance explained by the corresponding principal component. We find that different discretizations generally increase the dimensionality of the system (due to the introduction of noise in the form of discretization error). In Figure 3 we can observe how the dimensionality of the discretized systems departs from the original dimensionality depending on the number of bins employed. Specifically, we find that the choice of a binary description with just two bins is a particularly good description of the covariances of the system, increasing the dimensionality of the system by just 1.32% of the original dimensionality. Therefore, for the rest of the paper we will employ a binary description of the variables of the system as described below.

In previous work (Aguilera et al., 2015) we have determined that the configuration of the oscillators in the assembly is the relevant variable for generating one or other type of behavior. Therefore, we define cell assemblies depending on the relative phase of the oscillators with respect to the mean phase of the Kuramoto network. We codify the state of the cell assembly with a string of three bits Θ:

(7) $$\Theta =\{\Theta_{1},\Theta_{2},\Theta_{3}\},\text{ where }\Theta_{i}=\{\begin{array}{ll} 1, & \text{if }sin(\theta_{i}-\theta_{m})>0\\ 0, & \text{otherwise} \end{array}$$

where $\theta_{m}=∠(\frac{1}{3}\sum_{i}e^{\text{i}\theta_{i}})$ is the mean phase of the system at a particular instant.

Analogously to cell assemblies of neurons, we may consider a constellation of changing synaptic weights as an assembly of synapses or “synapsemble” (Buzsáki, 2010, p. 372). Synapse assemblies have been hypothesized to be critical for building up and dissolving cell assemblies and linking together sequences of cell assemblies. To codify the activity of synapse assemblies, we define each synapse as active or inactive if the value of the synapse is higher^3^ than the mean value of the synaptic strengths *K*_*m*_, where $K_{m}=\frac{1}{6}\frac{1}{T}\sum_{t}\sum_{i,j}K_{i,j}(t)$. The state of the synaptic assembly Ψ is codified with a string of 6 bits:

(8) $$\begin{array}{l} \Psi =\{\Psi_{1,2},\Psi_{2,1},\Psi_{1,3},\Psi_{3,1},\Psi_{2,3},\Psi_{3,2}\},\text{where}\\ \Psi_{i,j}=\{\begin{array}{ll} 1, & \text{if }K_{ij}>\langle K_{m}\rangle \\ 0, & \text{otherwise} \end{array} \end{array}$$

Similarly, for each trial we define a variable Λ which represents the behavioral pattern of the agent (i.e., what light it reaches):

(9) $$\Lambda =\{\begin{array}{ll} 1, & \text{if }d_{f,A}<d_{f,B}\\ 0, & \text{otherwise} \end{array}$$

where *d*_*f*, *A*_ and *d*_*f*, *B*_ are the final distances to each type of light at the end of the trial.

In summary, we define whether a particular cell or synapse ensemble is active by using a set of binary variables *s* = {Θ, Ψ, Λ} which represent if a specific ensemble is active at a particular moment of time and what behavioral pattern is being developed by the agent. All possible relations between oscillators give rise to 6 possible states for the cell assemblies, and all possible activated synapses give rise to 64 possible combinations or synapse assemblies, giving us a complete and discrete definition of the system that we can use to apply information theory tools. In order to do so 100 similar agents with random initial conditions are simulated for the situated and passively-coupled cases for 1000 trials with a duration of 1250 steps, generating a time series with 1.25 · 10^6^ states. Sections 3.2 and 3.3 use one of these time series to compute mutual information through time and transfer entropy, although the other 99 series yield practically identical results. The calculations in Section 3.1 require us to accurately compute the whole probability density function of the system. Thus, in order to avoid correlations in our sampling we compose 100 series of 10^6^ states, consisting in 10^4^ random states extracted from each of the initial 100 time series. Each sample can provide an estimation of the frequency of the 2^10^ states of *s*, inferring the probability density function of the system, which we compute as *P*(*s*) = *n*_*s*_/*n*_*T*_, where *n*_*s*_ is the number of occurrences of state *s*, *n*_*T*_ is the total length of the sample.

### 2.4. Information theory tools

Having defined these variables of the system in a discrete manner, we can use information theory tools to determine the relation between variables. In our model, we can use these measures to quantify the relation between the state of different elements in the neural controller of the agent, or between such components and features of the environment surrounding the agent. The information contained in a random variable is quantified in terms of *entropy*, which is defined as:

(10) $$\begin{array}{l} H\left(X\right)=-\underset{x\in X}{\sum }P\left(x\right)log\left(P\left(x\right)\right) \end{array}$$

where *X* is the set of states of the variable and *P*(*X*) its density probability function.

A useful measure to compare two variables is the relative entropy or Kullback-Leibler divergence between their statistical distributions, which is a measure of the difference between two probability distributions *X* and *Y*. It is defined as:

(11) $$\begin{array}{l} D\left(X;Y\right)=\underset{x\in X}{\sum }\underset{y\in Y}{\sum }P\left(x\right)log\frac{P\left(x\right)}{P\left(y\right)} \end{array}$$

Given a pair of variables *X*, *Y* and their marginal distributions the Kullback-Leibler divergence can be used to capture the information shared between the variables, defined as their *mutual information*:

(12) $$\begin{array}{l} I\left(X;Y\right)=H\left(Y\right)-H\left(Y|X\right)=\underset{x\in X}{\sum }\underset{y\in Y}{\sum }P\left(x,y\right)log\frac{P\left(x,y\right)}{P\left(x\right)P\left(y\right)} \end{array}$$

By definition, *I*(*X*; *Y*) = *I*(*Y*; *X*), thus mutual information cannot describe relations of causality. Instead, transfer entropy measures are typically employed to analyse causal relationships between variables. The decrease of uncertainty in the state of a variable derived from the past history of other variables is defined as the *transfer entropy* between two variables:

(13) $$\begin{array}{l} \begin{array}{c} TE\left(X\to Y\right)=H\left(Y_{t+\tau }|Y_{t}^{\left(d^{\prime }\right)}\right)-H\left(Y_{t+\tau }|Y_{t}^{\left(d^{\prime }\right)},X_{t}^{\left(d\right)}\right)= \end{array}\\ =\underset{x_{t+\tau },x_{t}\in X}{\sum }\underset{y_{t}\in Y}{\sum }P\left(x_{t+\tau },x_{t}^{\left(d\right)},y_{t}^{\left(d^{\prime }\right)}\right)log\frac{P\left(x_{t+\tau },x_{t}^{\left(d\right)},y_{t}^{\left(d^{\prime }\right)}\right)P\left(x_{t}^{\left(d\right)}\right)}{P\left(x_{t+\tau },x_{t}^{\left(d\right)}\right)P\left(x_{t}^{\left(d\right)},y_{t}^{\left(d^{\prime }\right)}\right)} \end{array}$$

where $X_{t}^{\left(d\right)}$ denotes the past history of *x* counted from time *t* and length *d* (i.e., *x*_*t*_, *x*_*t*−1_, …, *x*_*t*−*d*_).

## 3. Results

In this section we present results comparing a situated and a passively-coupled agent using a discrete description of the system and information theory tools. We first show that when the system is coupled to its environment it is brought closer to a regime of criticality and that the number of metastable states of the system is extended. These extended metastable states do not arise from the brain nor the agent in isolation but from the whole brain-body-environment system. We then use tools of mutual information to observe how neural plasticity in the agent in coordination is key for generating the structures that sustain flexible and metastable behavioral patterns. Finally, we use transfer entropy to characterize the loop of interactions between oscillatory dynamics, synaptic plasticity and behavioral patterns, describing the circular multiscale relation between the agent's neural controller and its behavioral dynamics necessary for generating extended sensorimotor metastable states.

### 3.1. Scale-free statistical distribution and mestastability

**Figure 4:**
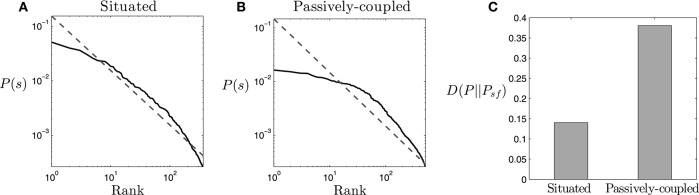
**Statistical distribution**. **(A,B)** Ranked probability density function of the states *s* = {Θ, Ψ, Λ} for **(A)** the situated agent and **(B)** the passively coupled agent (solid line), compared to the distribution of a Zipf-like distribution (dashed line). **(C)** Divergence between the probability density function of each type of agent and a Zipf-like distribution. States with probability lower than 2 · 10^−4^ are dismissed from the plot and calculations.

**Figure 5:**
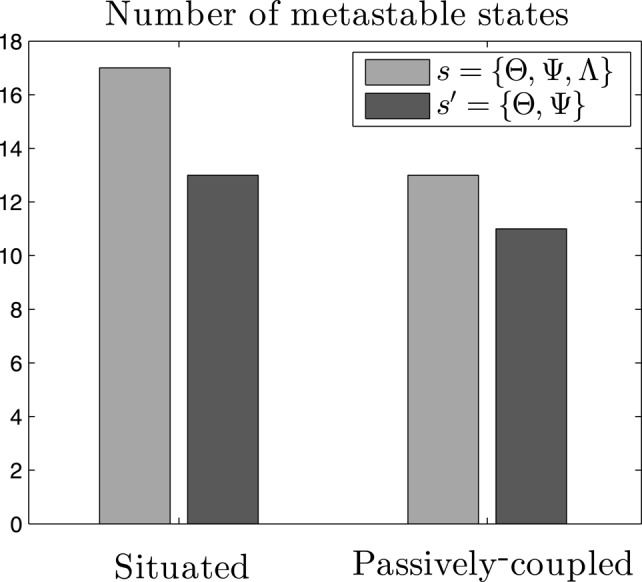
**Metastable states in the system**. Count of metastable states of the probability density function of neural and behavioral patterns *P*(*s*) (*s* = {Θ, Ψ, Λ}), and of neural patterns alone *P*(*s*′) (*s*′ = {Θ, Ψ}), for both the situated and passively-coupled agents.

We start by analysing the properties of the statistical distribution of the situated and passively-coupled agents. We compute the probability density function *P*(*s*) for each agent, where *s* = {Θ, Ψ, Λ}, by sampling 100 simulations of identical agents with random initial conditions during 1000 trials (1.25 · 10^6^ steps). We generate 100 samples, each one composed of 10^4^ random states *s* randomly sampled from each simulation run (10^6^ states in total) and calculate the frequency of occurrence of each state. The result from one randomly selected sample is shown in Figures 4, 5, while in the text we provide the statistics of the complete set of 100 samples.

An initial finding about the probability density function of the agent is that it approximately follows the Zipf law (Figure 4A), for states with a probability larger than 2 · 10^−4^. If the occurrence of the states of the system *s* is ordered by their decreasing frequency *P*(*s*), Zipf's law states that *P*(*s*) decays as the inverse of their rank *r*(*s*) in the ordered sequence, making *P*(*s*) ∝ 1/*r*(*s*). The occurrence of Zipf-like distributions is considered to be a signature of criticality (Mora and Bialek, 2011), coinciding with previous analysis of self-organized critical patterns in the same robotic agent when it is coupled with its environment (Aguilera et al., 2015). We observe that, while the situated agent presents a pattern very close to Zipf's distribution, the passively-coupled agent diverges more from a perfect scale-free distribution (Figure 4B), especially in states with higher probabilities. We can quantify this divergence by computing the Kullback-Leibler divergence between the distribution of states of the agent *P*(*s*) and Zipf's law distribution *P*_*sf*_(*s*) ∝ 1/*r*(*s*). As we observe in Figure 4C the divergence from the Zipf distribution in the passively-coupled agent is more than twice that of the situated agent. Computing the average and standard deviation for the 100 generated samples reveals that this result is repeated for different agents, confirming that the Kullback-Leibler divergence to a Zipf distribution is much larger in the passively-coupled agent (μ = 0.381, σ = 8.44 · 10^−4^) than in the situated case (μ = 0.141, σ = 5.46 · 10^−4^).

Criticality in the brain is generally associated with the metastability of transiently formed neuronal assemblies (Werner, 2007b), although in general the exact relation between the existence of metastable states and criticality is still not well understood. The definition of a metastable state is a state whose energy is lower than any of its adjacent states while not being the state of minimum energy of the system. If we assume that the probability of each state follows a Boltzmann distribution^4^, metastable states will be those with higher probabilities than those of their adjacent states. We define adjacency between two states when they are separated by a single flip^5^ of an individual variable Θ_*i*_, Ψ_*i*, *j*_ or Λ. In short, we consider metastable states as local peaks in the probability landscape. If we compute the number of metastable states of the system *s* = {Θ, Ψ, Λ}, we observe that the situated agent presents 17 metastable states for most of our data series (μ = 17.2, σ = 0.782)), while the passively coupled agent presents typically over 13 metastable states (μ = 13.1, σ = 0.902), indicating that the level of metastability is boosted when the agent is in interaction with its environment. However, if we only analyse the neural system of the agent, i.e., the system *s*′ = {Θ, Ψ}, we find that the situated agent presents 13 metastable states (μ = 13, σ = 0) and the passively-coupled agent presents around 11 (μ = 11.0, σ = 0.243). Figure 5 portrays the number of metastable states of one random sample.

These results show how the critical scaling and the repertoire of metastable states of the agent is extended when it is coupled with its environment. Moreover, metastable states generated when coupled with the environment cannot be reduced to metastable states in the “brain” of the agent (i.e., in *s*′ = {Θ, Ψ}) but only appear when we analyse the distribution of the complete system (*s* = {Θ, Ψ, Λ}). These results suggest that, aside from neural metastable states generated by oscillatory dynamics and neural plasticity, sensorimotor metastable states can appear from the coordination between behavioral patterns and internal neural dynamics. Spin glass theory indicates that metastable states emerge when some of the couplings between variables are negative (Mezard et al., 1987), which can be translated to stating that in our case agent-environment effective coupling presents mechanisms of mutual inhibition between pairs of variables. The appearance of metastable states only existing for the situated case in the whole sensorimotor system suggests a complex regulation between neural and sensorimotor processes, inviting us to take a closer loop of how agent-environment relations take place in order to increase the metastability of the system.

### 3.2. Mutual information flows

Typically, features such as criticality and metastability have been linked to the idea of systems driven by interaction dynamics between its components (Jensen, 1998; Van Orden et al., 2003; Ihlen and Vereijken, 2010). We investigate what type of interaction takes place in our agent between oscillatory dynamics, neural plasticity and behavioral patterns to generate neural and sensorimotor metastable states. We use information theory tools to quantify the interactions between the components of the system in the situated and passively-coupled agents, simulated for 1000 trials with a duration of 1250 steps. Although we only show the results of the time series of an individual simulation (random sampling is not applicable if we want to maintain temporal correlations), the differences in the results analyzing different runs of the simulation were negligible.

**Figure 6:**
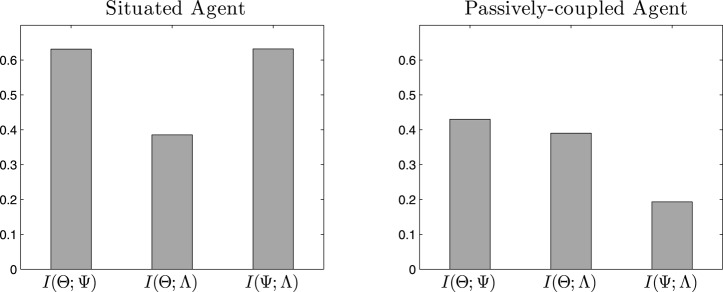
**Mutual information**. Values of mutual information between cell assemblies (Θ), synaptic assemblies (Ψ) and behavioral patterns (Λ) for the situated agent (left) and passively-coupled agent (right).

First, we analyse what information is shared by the emergent cell assemblies Θ, synapse assemblies Ψ and behavioral patterns of the agent Λ by measuring mutual information along the time series of values of each variable. In Figure 6 we can observe how the three variables share an important amount of information. The entropy of Λ (which is the variable with the lowest entropy) is 0.86, thus the shared entropy is in the same order of magnitude in most cases. In the case of the situated agent, we can observe in Figure 6 (left) that all variables share a relevant amount of information. However, in Figure 6 (right) we observe that the information shared between Ψ and Λ decreases dramatically, suggesting that most of the interaction between the two variables is lost.

The information analysis above shows a static picture of information flows on average, but it does not explain how these flows unfold over time. To overcome this limitation, Beer and Williams (2015) have proposed a framework combining information flow and dynamical analyses, exploring how a simulated model agent in a relational categorization task integrates information at different moments of time about a cue used for solving the task. Instead of analysing information as an average of the dependences between variables along a time series, they run the same task several times for different initial conditions and compute information measures for each time instant. Instead of using a series of temporal values of a variable, they use a series of values of a variable on each instant along different starting conditions. Similarly, we fold our time series into 1000 time series (one for each simulated trial) in which e.g., $\Theta_{T}^{\prime }\left(t\right)=\Theta \left(t+\left(T-1\right)1250\right)$, where *T* is the trial number and 1250 is the duration of each trial. Consequently, for each value of *t* we have 1000 values of $\Theta_{T}^{\prime }\left(t\right)$ we can use for computing mutual information with other variables. For six consecutive trials (i.e., *t* = 1, …, *t*_*end*_ = 6 · 1250) we compute the mutual information between the neurodynamic variables of the agent $\Theta_{T}^{\prime }\left(t\right)$ and ${\text{Ψ}}_{T}^{\prime }\left(t\right)$ and the behavioral pattern at the end of the sixth trial $\Lambda_{T}^{\prime }\left(t_{end}\right)$ (in order to observe how information about future behavioral patterns is accumulated). Also, we compute the joint mutual information that both $\Theta_{T}^{\prime }\left(t\right)$ and ${\text{Ψ}}_{T}^{\prime }\left(t\right)$ share with $\Lambda_{T}^{\prime }\left(t_{end}\right)$.

**Figure 7:**
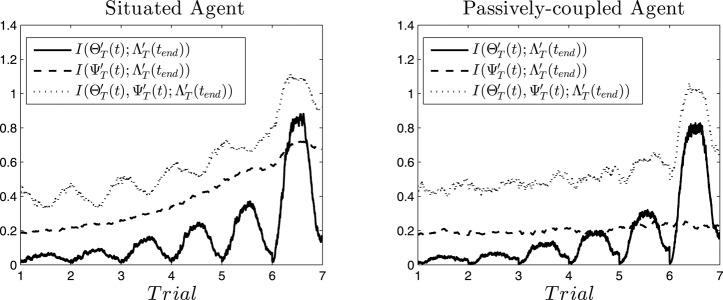
**Information flows**. Mutual information at different instants between cell assemblies ($\Theta_{T}^{\prime }\left(t\right)$), synaptic assemblies (${\text{Ψ}}_{T}^{\prime }\left(t\right)$) and the behavioral patter displayed at the end of the sixth trial ($\Lambda_{T}^{\prime }\left(t_{end}\right)$) for the situated agent (left) and passively-coupled agent (right).

In Figure 7 (left) we can observe the result for the situated agent. We see how the mutual information $I\left(\Theta_{T}^{\prime }\left(t\right);\Lambda_{T}^{\prime }\left(t_{end}\right)\right)$ increases during the middle of the trial, fading out at the beginning and the end. That is, the activation of specific neural patterns when the robot is approaching a light contributes to its repetition in future trials (i.e., contributing to the habit of choosing that light). However, when the robot engages in other behavior (e.g., exploring its surroundings, or stopping after having reached a light) the habit is no longer being enacted and the information about it vanishes from its oscillatory patterns. Also, from one trial to the next the information at the peak increases, being maximal at the sixth trial. We can interpret this as a self-sustaining behavior of cell assemblies: when a cell assembly emerges, it reinforces itself and has more probabilities to reemerge in the next trial. Similarly, $I\left({\text{Ψ}}_{T}^{\prime }\left(t\right);\Lambda_{T}^{\prime }\left(t_{end}\right)\right)$ steadily increases until a cell assembly is activated at the middle of the sixth trial. Mutual information between ${\text{Ψ}}_{T}^{\prime }\left(t\right)$ and $\Lambda_{T}^{\prime }\left(t_{end}\right)$ is continuously accumulated and does not decrease, thus we hypothesize that the configuration of the synapse ensembles “stores” information about the behavior that the agent will develop. Furthermore, when we analyse the joint mutual information $I\left(\Theta_{T}^{\prime }\left(t\right),{\text{Ψ}}_{T}^{\prime }\left(t\right);\Lambda_{T}^{\prime }\left(t_{end}\right)\right)$, we observe that it is always higher that the individual contributions. Moreover, it increases when $I\left(\Theta_{T}^{\prime }\left(t\right);\Lambda_{T}^{\prime }\left(t_{end}\right)\right)$ decreases at the transitions between one trial and another. Also, $I\left(\Theta_{T}^{\prime }\left(t\right),{\text{Ψ}}_{T}^{\prime }\left(t\right);\Lambda_{T}^{\prime }\left(t_{end}\right)\right)$ decreases when at the middle of the trial a cell assembly is activated, except in the last trial. This portrays an interesting picture, where information flows back and forth between the emergent cell assemblies and the collective cell-synapse assemblies, until the sixth trial when an assembly emerges producing behavior $\Lambda_{T}^{\prime }\left(t_{end}\right)$.

If we analyse the passively-coupled agent we observe a quite different picture (Figure 7, right). Although $I\left(\Theta_{T}^{\prime }\left(t\right);\Lambda_{T}^{\prime }\left(t_{end}\right)\right)$ is quite similar in both cases (its values are slightly smaller in the passively-coupled condition), $I\left({\text{Ψ}}_{T}^{\prime }\left(t\right);\Lambda_{T}^{\prime }\left(t_{end}\right)\right)$ does not integrate any information. This suggests that even when the input produced by behavior $\Lambda_{T}^{\prime }\left(t_{end}\right)$ is able to influence the cell assemblies that emerge, coordination between behavior and the stabilization of synapse assemblies does not take place. Furthermore, the joint information $I\left(\Theta_{T}^{\prime }\left(t\right),{\text{Ψ}}_{T}^{\prime }\left(t\right);\Lambda_{T}^{\prime }\left(t_{end}\right)\right)$ does not integrate much information either, and the anticorrelation between $I\left(\Theta_{T}^{\prime }\left(t\right);\Lambda_{T}^{\prime }\left(t_{end}\right)\right)$ and $I\left(\Theta_{T}^{\prime }\left(t\right),{\text{Ψ}}_{T}^{\prime }\left(t\right);\Lambda_{T}^{\prime }\left(t_{end}\right)\right)$ disappears. This suggests that the passively-coupled agent does not capture the struggle between information flows through individual and collective variables, indicating that the important moments for generating the behavior of the agent are not only synchronizing moments of emergence of cell assemblies, but that most information is built during instants of desynchronization corresponding to transition from one assembly to the next.

These results show that synaptic plasticity, in coordination with behavioral patterns, plays a fundamental role in the situated agent, since it allows the agent to “store” information about future behavioral patterns the agent will engage it. Interestingly, the results in Figure 7 suggest that neural assemblies behind the execution of a specific behavioral pattern reinforce the synaptic circuits sustaining that pattern, which store information about the repetition of that behavioral pattern. This resonates with the idea of the brain as a plastic system of open loops (Fuchs, 2011) created during previous interactions with the environment and functionally closed to full sensorimotor cycles in every new coupling with the environment. Sensorimotor metastable states could be precisely the transient closure of those loops, which in turn imprint and reinforce onto the brain the synaptic structures necessary to their reproduction.

### 3.3. Transfer entropy

**Figure 8:**
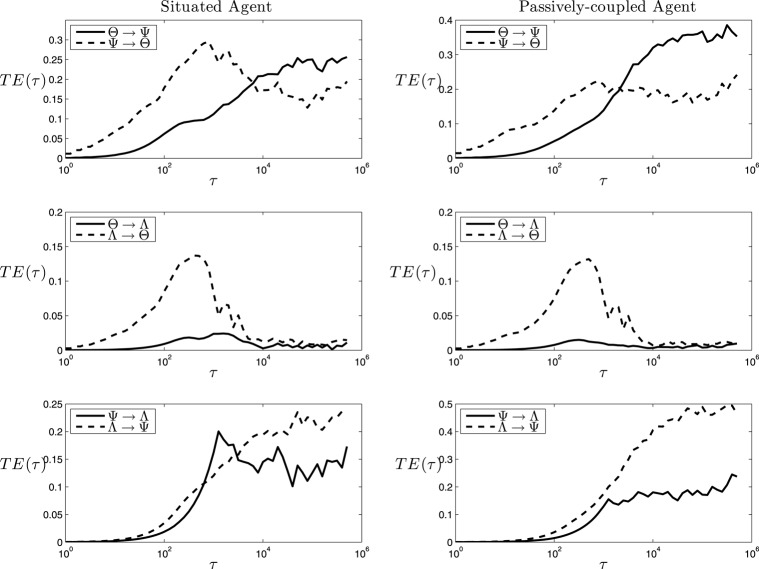
**Transfer Entropy**. Values of transfer entropy at different timescales among cell assemblies (Θ), synaptic assemblies (Ψ) and behavioral patterns (Λ). Note that the duration of a trial corresponds to a value of τ of 1250 steps.

The analysis above shows the information shared by variables unfolding through time. As mutual information is a symmetric index, it is not a good tool to characterize causal interactions between parts of a system. Instead, we characterize directional interactions by measuring transfer entropy between variables using Equation 2.4, with *d* = *d*′ = 1 as the length of the past history that we take into account^6^ and a logarithmically distributed series of values of τ from 1 to 625, 000 steps (half the length of the 1000 trials), with multiplicative intervals of 10^0.1^. In Figure 8 (left) we can observe a complex chart of information flows for the situated agent:

- Θ − Ψ transfer. We can observe transfer entropy from Θ to Ψ taking place at small and medium values of τ, whereas at larger values of τ the flow of the information is reversed. This suggests a circular causal chain in which, at short timescales, the structure of the current synaptic assembly determines the cell assemblies that can emerge, but at long timescales it is the self-sustainment of particular assemblies during different trials that determines the stability of the possible synaptic assemblies.
- Θ − Λ transfer. We observe that while there is an important transfer entropy flow from Λ to Θ at fast and medium timescales, the flow does not exist in the opposite direction (Figure 8, middle-left), suggesting that the behavioral pattern of the agent influences the cell assembly that emerges, but that the current cell assembly that is active at a particular moment of time is not decisive for the behavioral pattern that the agent will deploy.
- Ψ − Λ transfer. There is an important bidirectional exchange of information between Ψ and Λ. This suggests that Ψ is the variable that determines the behavior that will be chosen by the agent. Also, we can observe that *TE*_Λ → Ψ_ and *TE*_Θ → Ψ_ are very similar in value and shape (if we integrate the area of the difference between *TE*_Λ → Ψ_ and *TE*_Θ → Ψ_ and divide it by *TE*_Θ → Ψ_ the result is 0.11, showing that both functions coincide with almost 90% of accuracy). This is supported by the fact that there is no functional dependency from Λ to Ψ, since change in the weights $\delta \dot{K}$ is only a function of *K* and θ. As we can easily check, all the other information flows present in Figure 8 correspond to actual functional dependencies depicted by the equations defining the systems^7^. This suggests (since Λ influences Θ and not otherwise), that Λ causally determines Θ which in turn influences Ψ in their circular mutual interaction^8^.

**Figure 9:**
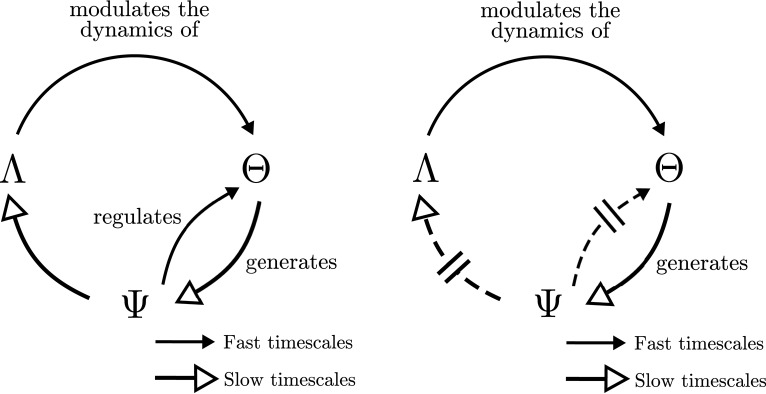
**Operational closure**. Simplified information flows among cell assemblies (Θ), synaptic assemblies (Ψ) and the behavioral patterns (Λ) for the situated (left) and passively-coupled (right) agents.

Putting together the transfer entropy flows above, we may summarize them in the schema at Figure 9 (left). The behavior of the agent Λ generates an input that determines the emergence of cell assemblies Θ at fast timescales. A circular relation between the emergent cell assemblies and their underlying synapse ensembles Ψ generates a particular behavior Λ which is determined by the state of Ψ at longer timescales. We can observe how the resulting schema is similar to the one proposed by Varela (1997) and depicted in Figure 1, though adding interesting information about the timescales of each dependency.

Moreover, for the passively-coupled agent, the information flows from Ψ to Λ and from Ψ to Θ are disrupted and reduced in comparison to other flows, whereas information flows from Λ to Θ and Θ to Ψ^9^ are maintained or even increased. This disruption of part of the transfer entropy flows dissolves the self-sustained neurodynamic structures that generate a coordinated behavior and reinforcing unidirectional influence from Λ to Θ and Θ to Ψ, as we depict at Figure 9 (right).

These results strongly suggest that the generation of complex and integrated neurodynamic structures is a product of a double circular loop that strongly couples (1) neural oscillatory patterns with the plastic synaptic structures sustaining them and (2) these neurodynamic circular structures with the behavioral patterns generating them. This double loop is constituted in a way that creates a circular asymmetry between agent and environment, in which the oscillatory dynamics of the agent present a sensitivity of environmental parameters at shorter timescales, and its repeated activation generates the synaptic structures that are able to engage and disengage different behavioral patterns of the agent at longer timescales. The operational closure of the nervous system implies a special circular relationship between agent and environment, in which the agent individuates itself in front of its environment as it is capable of being sensitive to small fluctuations of its world while being able to act over it at longer timescales as a coherent dynamical unit.

## 4. Discussion

In this paper we have presented a neurodynamical model of oscillatory activity with synaptic activity embedded in a robotic agent in a behavioral preference task. The model is based on a network of three Kuramoto oscillators with plastic homeostatic mechanisms designed to maintain constant phase relations among oscillators. Our goal was to explore metastability in behavior and neural assemblies in a context of embodied, adaptive activity, in which the agent is in continuous and bidirectional interaction with an environment, a dimension which is frequently neglected in the study of brain activity and organization. The model was carefully designed for exploring (1) the integration of transient assemblies underlying behavior through nonlinear coupling neural clusters generating specific conducts in the agent, and (2) the coordination between sensorimotor and plastic neurodynamic structures into a self-maintaining behavioral patterns. The integration of these two levels of activity gives rise to metastable sensorimotor integrated patterns which cannot be reduced to metastability of brain dynamics alone, as behavioral preferences of the agent emerge from the interaction between oscillator cell ensembles, ensembles of synaptic weights and the agent's sensorimotor coupling. We present a methodological framework to analyse the role of sensorimotor behavior in interaction with neural dynamics: we compare a situated agent, normally interacting with its environment, and a passively-coupled agent, receiving a sensory input recorded from the situated agent but unable to influence its environment in any way. Comparing a passively-coupled agent and a situated agent in an open environment we have found three different results shedding light on the relation between neural and behavioral metastability.

These results are obtained first, through a statistical description of the agent (in the form of a discrete characterization of the states of the agent neural configurations and behavioral patterns), depicting how the situated agent presents signatures of criticality and additional metastable states that are not present in the passively-coupled agent. Moreover, we show how those additional metastable states do not appear in neural variables alone, but in the combined space of neural and behavioral patterns, indicating that metastable states in behavior are not exclusively a direct mapping of neural metastable states. Instead, we find the existence of sensorimotor metastable states that extend the range of metastability of the agent's “brain.”

Second, an analysis of the flow of mutual information between different groups of variables of the agent shows that in both the situated and passively-coupled models neural ensembles of oscillatory components contain a lot of information about the behavioral pattern being developed. However, this information is lost after the neural ensemble dissolves at the end of a trial, and only in the situated case this information is stored instead through changes in synaptic plasticity, generating the structures reinforcing future appearances of that behavioral pattern. This suggests that mechanisms of synaptic plasticity have a fundamental role in coordinating neural and behavioral processes, sculpting the sensorimotor structures sustaining extended metastable states. While synapse assemblies have been hypothesized to have a critical role for building up and dissolving metastable cell assemblies in the brain as well as linking together sequences of cell assemblies (Buzsáki, 2010, p.372), our results invite us to rethink the role of synapse assemblies in a broader sense, as fundamental elements that facilitate emergence and dissolution of metastable modes of engagement with the environment.

Finally, using transfer entropy we can depict the causal influences at different timescales between different components of the system. We observe how the situated agent generates a closed network of interaction, circularly organized with bidirectional interactions at different timescales. This network takes the form of a double coupling loop of (1) a circular causal dependence between the emergence of cell assemblies and the synaptic neural structure that generates them, and (2) a causal chain in which synaptic structures influence the behavior displayed by the agent, which in turn triggers the emergence of specific neural assemblies (Figure 9, left). If the sensorimotor loop is disrupted (e.g., when the agent is passively-coupled, and probably for more severe sensorimotor disruptions), this circular closure disappears and autonomous organization of the agent vanishes (Figure 9, right). This provides cues of what happens in real-life examples of disruption of sensorimotor coordination. For example, in Held and Hein's experiment on visually-guided behavior (Held and Hein, 1963), the “passive” kitten fails to develop perceptual abilities. Similar situations can take place in different physiological or pathophysiological conditions. Examples of those are the problems faced by deafferented subjects (i.e., without any proprioception) to develop behavioral automatisms exclusively in the absence of sensory feedback; needing to rely on visual feedback to perform simple tasks such as holding an egg without breaking it, or when are unable to maintain an upright posture in the dark (Cole and Paillard, 1995).

Interestingly, the schema of circular dependencies in the agent resembles the idea of operational closure of the nervous system proposed by Varela (1997) depicted in Figure 1. Moreover, it describes a novel characteristic of closure since this loop of closure creates a multiscale asymmetry between agent and environment: the agent is sensitive to changes in the environment at fast timescales, while it can influence the environment at slow timescales (Figure 8, left). Although the agent's oscillatory dynamics are mostly driven by inputs from the environment at fast timescales, it exerts an influence over the environment at the slower timescales of synaptic plasticity by generating structured behavioral habits (e.g., reaching repeatedly one of the lights) which will influence future stimuli received by the agent. In some sense, once the agent “sees” a light it is trapped in a behavioral field and has to reach it, but it still has a degree of autonomy in the sense that it can modulate its internal connectivity to influence which lights it is going to be sensitive to in the future. This allows us to identify the agent as a unit which is affected by bottom-up causal flows of sensorimotor stimuli and, at the same time, it is able to develop a downward causation modulating its sensorimotor interaction. Breaking the symmetry of the coupling between agent and environment has been proposed as one of the fundamental aspects which can constitute an agent as an autonomous entity able to regulate from within its exchange with the world, constituting its identity as a self-individuating system (Barandiaran et al., 2009). Previous characterizations of agent-environment asymmetry have referred only to the presence of a directionality in the flows of information from agent to environment depicting a causal influence from the former to the latter (see Seth, 2007; Bertschinger et al., 2008). These contributions do not take into account a self-referential operational closure of the system (Bertschinger et al., 2008, p.14) and only quantify the degree of self-determination of the system. In contrast, our approach captures agent-environment asymmetry as a circular relation of causal influences at different scales.

The type of analysis performed in our model has typically been unexplored by neuroscientists studying real life organisms, partly due to the difficulty of recording whole-brain activity of freely behaving animals. In general, recordings of neural activity have been limited to either small brain regions or to immobilized or anesthetized animals exhibiting limited behavior. Nonetheless, during the last few years some promising results point to the plausability of experiments involving the sensorimotor engagement of whole-brain or large brain areas. For example, an interesting technique for analysing brain and behavioral activity of a head-restrained mouse interacting with a virtual reality environment in a spherical treadmill (see Dombeck et al., 2007) has been developed. Furthermore, the first report of whole-brain recording in freely behaving animals has been reported for the nematode Caenorhabditis elegans during free locomotion (Nguyen et al., 2016). These advances open-up an exciting path for neuroscience, allowing an exploration of how interesting properties of neural processes such as criticality and metastability are extended and amplified when they are embedded in ongoing embodied sensorimotor loops. In such scenarios, minimal models of brain-body-environment dynamical regulation in adaptive behavior, such as the one presented here, offer a conceptual basis for facing complex analysis in real animals due to the low dimensionality of their dynamics. Even models that have little connection with biological brains can provide insights into how neural and sensorimotor dynamics may interact (e.g., extending the range of metastable states, creating asymmetrical loops of causal interaction, etc.), as well as contribute to the development of conceptual methodological tools for understanding the role of the different scales of the system (e.g., the situated vs. passively-coupled comparison to address the role of sensorimotor regulation in the nervous system). Moreover, the availability of real data of brain-body-environment interaction will provide the opportunity to advance in the design of more accurate and realistic models, bringing us closer to capturing fundamental aspects of adaptive behavior.

## Author contributions

Conceived and designed the experiments: MA, MB, XB. Performed the experiments: MA. Analyzed the data: MA. Wrote the paper: MA, MB, XB.

## Funding

This research has been partially supported by the project PSI2014-62092-EXP of the National Programme for Fostering Excellence in Scientific and Technical Research (“Explora Ciencia” call) from the Spanish Ministry of Economy and Competitiveness.

### Conflict of interest statement

The authors declare that the research was conducted in the absence of any commercial or financial relationships that could be construed as a potential conflict of interest.

## Footnotes

^1^Phototaxis implies climbing a light gradient. This task has been chosen because gradient climbing is a minimal task, which is widespread in nature. Many small scale adaptive behavior occurs along chemical gradients, and the microscopic world is full of gradients (like thermal gradients or light gradients but mostly chemical gradients). The adaptive behavior of small animals (e.g., C. elegans) and individual motile cells (e.g., bacteria but also animal cells migrating during development) is mostly a gradient-related adaptive behavior. Navigating smell or heat gradients are also stereotypical adaptive tasks for higher animals. Moreover many instances of higher-level behavior can also be interpreted as abstract gradient climbing (e.g., a human can move up a gradient of social popularity or economic wealth involving complex strategic decisions).

^2^This continuous, non-monotonic change of synaptic strengths was introduced by Iizuka and Di Paolo (2008) as a simple mechanism to avoid weight saturation of Hebbian-like rules. This way, eschewing biological plausibility allows us to make sure that the robots is able to explore its whole range of weight configurations.

^3^*K*_*m*_ is an arbitrary threshold for capturing when weights are active and sufficiently strong. Different values have been tested without altering the results.

^4^The situated system can be considered an isolated system in thermodynamic terms, whereas the passively-coupled system may be considered a closed system exchanging energy but not matter with the situated system, thus making plausible the assumption of thermal equilibrium and a Boltzmann distribution of states, and therefore a direct mapping between probabilities and the energy of each state.

^5^Other definitions of metastability could be used, using an arbitrary number *n* of flips. Nevertheless, in this particular case only *n* = 1 yields the existence of mestastable states.

^6^We tested different values of *d* and *d*′ up to 5 for a series of representative values of τ with similar results, therefore we used *d* = *d*′ = 1 to reduce the computational cost, since just varying the value of τ seems to be enough for capture the different timescales of information flows.

^7^Since $\dot{\theta }$ is a function of θ, *K* and *s*_*DX*_, and ṡ_*DX*_ is a function of θ, *K* and *s*_*DX*_. The only functional dependency that presents no informational content is *TE*_Θ → Λ_

^8^It is worthy noting that in the experiments depicted in Figure 8, the variable Λ is defined as the macroscopic behavior of the agent, whereas Θ and Ψ are patterns of low level variables of the agent. To test that this difference did not distort in any way the result of our analysis, we repeated the analysis substituting Λ by a variable *S* defined as a string of 4 bits (one for each sensor of the robot), in which each bit was equal to one if the activity of its corresponding sensor is higher than its average activation. The results of transfer entropy measures were strikingly similar to those displayed in Figure 8, indicating that the series of sensory activation are strongly correlated with particular behavioral macroscopic patterns of the agent. Since the use of Λ simplifies other analyses performed in this paper, we have chosen to use this variable instead of *S*.

^9^Flows from Θ to Ψ are almost equivalent to flows from Λ to Ψ, supporting our suspicion that they represent the same information flow.
